# Comprehensive surgical treatment strategy for spinal metastases

**DOI:** 10.1038/s41598-021-87121-1

**Published:** 2021-04-12

**Authors:** Arthur Wagner, Elena Haag, Ann-Kathrin Joerger, Philipp Jost, Stephanie E. Combs, Maria Wostrack, Jens Gempt, Bernhard Meyer

**Affiliations:** 1 Department of Neurosurgery, Klinikum Rechts Der Isar, Technical University Munich School of Medicine, Ismaninger Str. 22, 81675 Munich, Germany; 2 Department of Hematology and Oncology, Technical University Munich School of Medicine, Munich, Germany; 3 Department of Radiation Oncology, Technical University Munich School of Medicine, Munich, Germany

**Keywords:** Cancer, Surgical oncology, Neurology, Prognosis

## Abstract

The management of patients with spinal metastases (SM) requires a multidisciplinary team of specialists involved in oncological care. Surgical management has evolved significantly over the recent years, which warrants reevaluation of its role in the oncological treatment concept. Any patient with a SM was screened for study inclusion. We report baseline characteristics, surgical procedures, complication rates, functional status and outcome of a large consecutive cohort undergoing surgical treatment according to an algorithm. 667 patients underwent 989 surgeries with a mean age of 65 years (min/max 20–94) between 2007 and 2018. The primary cancers mostly originated from the prostate (21.7%), breast (15.9%) and lung (10.0%). Surgical treatment consisted of dorsoventral stabilization in 69.5%, decompression without instrumentation in 12.5% and kyphoplasty in 18.0%. Overall survival reached 18.4 months (95% CI 9.8–26.9) and the median KPS increased by 10 within hospital stay. Surgical management of SMs should generally represent the first step of a conclusive treatment algorithm. The need to preserve long-term symptom control and biomechanical stability requires a surgical strategy currently not supported by level I evidence.

## Introduction

Metastases to the spinal column have developed considerable epidemiological significance in the recent decades^1–3^. Advances in standards of care and targeted systemic therapies constituted a substantial increase in life expectancy, which in turn has led to a reassessment of treatment strategies^4^. Surgical treatment has thus been upgraded to an indispensable instrument within the overall treatment algorithm. Pioneered by Patchell et al. in 2005, surgical management has since evolved from simple decompression in cases of acute neurocompression to the key first step in the overall interdisciplinary management of spinal metastases (SMs)^3,5–8^. The aforementioned growing life expectancy sees patients in need of long-term pain relief, functional independence and a stable spine, putting emphasis on surgical stabilization procedures. Simultaneously, the advent of minimally-invasive approaches, routine use of navigational systems and specialized pedicle screw systems for improved adjunct treatment planning have facilitated drastically improved safety and efficacy^9–14^. The gold standard of surgical treatment of SMs, a posterior instrumentation with decompression, is no longer reserved to a limited selection of patients. Simultaneously, available surgical strategies have been developed to incorporate techniques for anterior column reconstruction, which facilitates a more invasive circumferential stabilization for selected cases. The addition of a corpectomy with vertebral body replacement may principally secure stabilization in the long-term, although evidence concerning this matter is still pending.

The SM’s innate heterogeneity is a product of vastly differing growth rates, biomechanical destabilization and systemic affection all of which are dictated by a multitude of primary entities. Most commonly, breast and prostate carcinomas preferably seed to the axial skeleton in up to 70% of postmortem examinations compared to a mere 10% for gastrointestinal cancers^15–17^. By nature, this heterogeneity challenges a cluster of interdisciplinary specialists involved in both surgical and oncological fields^3,8^. For every individual patient, it is now regarded mandatory for specialized oncological care centers to adopt a stratified treatment algorithm, integrating surgical instrumentation, stereotactic radiation as well as targeted systemic therapy regimens based on a number of determinants. Naturally, there may be considerable differences in treatment principles between oncological care centers, which renders any direct comparison of outcomes inapt.

Following this principle, the aim of our study was to report a sizable consecutive cohort of patients with SMs managed in accordance with a treatment algorithm of a single oncological cancer center. To our knowledge, this represents the largest surgically treated single-center consecutive cohort of patients with SMs reported in literature so far.

## Methods

### Patient population

We analyzed our database of consecutive patients treated for newly diagnosed SMs at our Department of Neurosurgery. Every patient was screened from the time a treatment plan was devised by a Neurooncological Board meeting consisting of neurosurgeons, radiation therapists, oncologists and radiologists for every individual case. The rationale behind recommending surgical management and further treatment or following alternative strategies was thus based on collective decision-making.

Assessments at the time of diagnosis included baseline demographics, comorbidities, neurological deficits and functional status gauged by the Karnofsky Performance Status Scale^18^ (KPS) at baseline and on follow-up.

The radiographic characteristics of SMs were used to determine the common prognostic indices and scores including the Spinal Instability Neoplastic Score^19^ (SINS), the revised Tokuhashi Score^20^ and the Tomita Score^21^. The assessment of these scores were individually reviewed by two independent neurosurgeons. There were no exclusion criteria applied to the population to allow for a generalizable cohort.

Patients were instructed to adhere to follow-up examinations in the outpatient clinic of the Cancer Care Center with imaging conducted at 3-month intervals. Regular staging imaging by computed tomography (CT) were used to assess disease progression. Treatment planning for adjunct irradiation of the SM was done by contrast-enhanced magnetic resonance imaging (MRI).

### Surgical treatment

The primary surgical treatment consisted of a posterior instrumentation with decompression. This decompression was primarily conducted from posterior in the same sitting via laminectomy of the affected segment(s), provided that a manifest epidural compression or radiculopathy was present. A vertebral body replacement was added depending on several factors: patients with rapidly progressing primary entities in a reduced general condition were scheduled for early adjunct radiation and initiation of chemotherapy. In cases of acutely compromised stability with anterior compression by an osteolytic SM and if the patient’s general condition allowed for it, an early staged vertebral body replacement was added before initiation of the adjunct therapy regimen. This approach was favoured over a total spondylectomy combined with separation surgery as advocated recently by numerous authors^8,22–24^.

Posterior instrumentation and decompression were performed preferably with a minimally-invasive technique, with navigated placement of pedicle screws routinely in every case. The decision for cement-augmentation was based on the biomechanical integrity of the cancellous bone judged on preoperative CT and bone mineral density scans, if available. Until 2015, the pedicle screw instrumentation was done with titanium alloy systems. Beginning in Juli 2015, we employed a polyaxial carbon-fiber-reinforced carbon/poly-ether-ether-ketone pedicle screw system fenestrated with cement-augmentation capabilities. After 2018, the system was suitable for minimally-invasive instrumentation.

This algorithm was altered only in select cases by verdict of the Neurooncological Board, mainly when a palliative care strategy had been decided upon and the patients were unfit for instrumentation procedures. Still, these patients were treated symptomatically for axial pain by kyphoplasty or for neurological compromise by sole decompression. A biopsy was taken for histopathological analysis in every case, irrespective of chosen surgical strategy.

These principles are applied to cervical, thoracolumbar and sacral SMs equally.

### Statistical analysis

For the primary outcome, a multiple linear regression was conducted for prediction of overall survival (OS) after 12 months as a function of preoperative independent variables and composite scores. Secondary analyses included binomial logistic regression analyses of independent predictors for mortality and clinical deterioration after surgery as well as multiple linear regression analyses of the postoperative KPS. For the Kaplan–Meier survival estimates, patients were stratified into subgroups according to their surgical procedure, their KPS at discharge and their ability to ambulate with or without walking aids by discharge. We used IBM SPSS in its 25^th^ (https://www.ibm.com/products/spss-statistics) version for statistics, the level of significance was defined a priori as *α* = *0.05*.

### Ethical considerations

All procedures were indicated and conducted in compliance with our department’s standards and the Declaration of Helsinki. The Ethics Committee Klinikum rechts der Isar of the Technical University Munich (Ethikkommission Klinikum rechts der Isar der Technischen Universität München) granted a positive vote (reference no. 96/19S) and waived the requirement for written informed consent.

## Results

### Epidemiology

A consecutive cohort of 667 patients undergoing 989 procedures was collected between January 2007 and December 2018. Demographic data and perioperative functional status were available for every case, preoperative imaging data was missing in two cases. Baseline and radiographic characteristics that factored into the regression analysis are listed in Table 1. Follow up data was available for 71.2% of patients, mean follow up amounted to 17.8 months (95% confidence interval [CI] 15.1–20.4 months). During follow-up, 182 patients (27.3%) had died.

**Table 1:** Baseline and radiographic characteristics of cohort.

Age in years, mean (range)	65 (20–94)
Sex: female	39.7%
Mechanical pain	83.5%
Neurological deficit	45.7%
Osteolytic lesion	83.2%
**Segmental deformity**	
Kyphotic or lordotic	23.1%
Subluxation	2.7%
SINS ^19^, mean (range)	9 (2–17)
Systemic metastases (excluding spine)	42.7%

**Figure 1:**
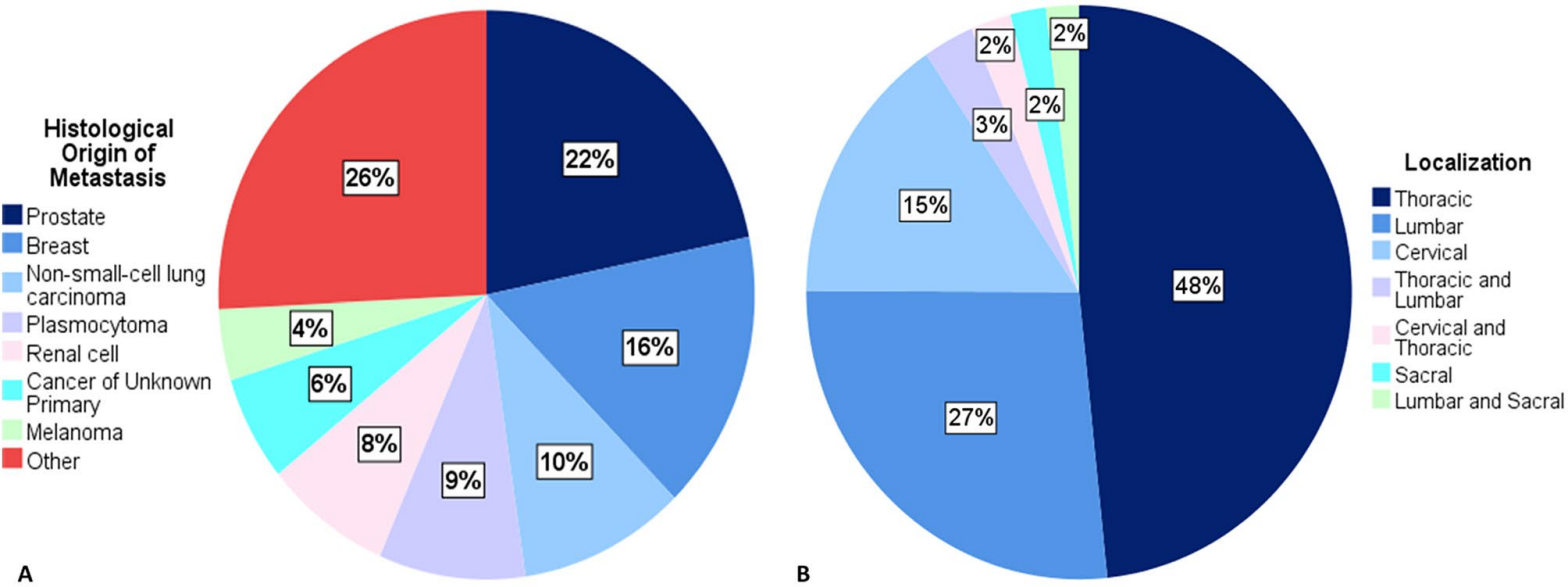
Distributions of primary cancer origin **(A)** and localization in the spine **(B)**. Other—colorectal, pharynx, hepatocellular, uterus, pancreas, sarcoma, lymphoma, gastric, urothelial, thyroid, testicular, parotid, small-cell lung, adrenal, duodenal, cholangiocellular.

The proportions of cancers of origin are depicted in Fig. 1A. A total 318 (47.6%) of SMs stemmed from prostate, breast or non-small-cell lung cancer. The majority of SMs (48%) were located in the thoracic spine, followed by the lumbar spine (22%). Multiple segments were affected in 7% of cases (Fig. 1B).

The means and ranges of the SINS score are listed in Table 1. For the SINS, 79.6% of patients had scores of at least 7, signifying potential or manifest instability. Further, 54.9% of patients had a score of at least 6 on the Tomita scale 86.1% had a score of 11 or less on the revised Tokuhashi scale, each representing an estimated survival of 12 months at most.

### Preoperative deficits and neurological outcome

On presentation, axial pain was noted in 557 patients (83.5%) and 29 (4.4%) were asymptomatic with an incidental finding of a SM. Any neurological deficit was found in 305 patients (45.7%), the rest were neurologically intact.

The most common form of neurological impairment was paresthesia located at the dermatome of the affected segment in 231 patients (34.6%). A motor palsy, defined as a motor deficit of grade 4 out of 5 or lower according to the Medical Research Council (MRC) scale, was noted in 205 patients (30.7%) on admission. Within the entire cohort, 17 patients (2.5%) exhibited a complete spinal cord injury corresponding to American Spinal Injury Association (ASIA) Impairment Scale grade A, while 7 patients (1.1%) had ASIA B.

Postoperatively, 592 patients (88.8%) noted a complete resolution of pain or a reduction of at least 2 on the Visual Analogue Scale, the rest experienced no notable change or an increase in pain intensity. Among those 205 patients with a motor palsy on admission, 145 patients (21.7%) improved by at least one MRC grade after surgery. For those 17 patients with a preoperative ASIA grade A, substantial improvement to ASIA C was seen in 8 patients (1.2%), improvement of anesthesia to ASIA B in one patient (0.2%), while the remaining 8 (1.2%) retained ASIA grade A. One patient (0.2%) with a preoperative ASIA B improved to ASIA D and 2 patients (0.3%) improved to ASIA C, while 4 (0.6%) did not improve.

Twenty-six patients (3.9%) without preoperative deficits experienced postoperative neurological deterioration. Of these, 19 deficits (2.9%) were transient and had fully recovered during the postoperative in-patient stay. The occurrence of postoperative neurological deterioration was not significantly influenced by age (*p* = 0.166), the surgical procedure (*p* = 0.692), the cancer of origin (*p* = 0.817) or the affected spinal segment (*p* = 0.995) in the logistic regression analysis.

The median preoperative KPS was 70, which increased to 80 at discharge, remaining 80 after 3 months and after 12 months. On admission, 69.6% of the entire cohort had a KPS equal or above 70. This proportion significantly increased to 72.7% postoperatively (*p* = 0.026), to 77.2% at 3 months (*p* = 0.041) and non-significantly to 83.0% at 12 months (*p* = 0.250). Thirty patients (4.5%) who presented with a KPS ≥ 70 deteriorated to a postoperative KPS < 70. In contrast, 51 patients (7.7%) improved from a preoperative KPS < 70 to ≥ 70 after the surgery. Between patients with neurological deficits on admission and without, there was a significantly higher proportion of males in the group with deficits (65.8% vs. 55.5%; *p* = 0.006). In the latter group without neurological impairment, both preoperative and postoperative median KPS were 80 (mean difference 0.4; *p* = 0.559), while the group with neurological deficits improved from a preoperative 60 to 70 by discharge (mean difference 3.9; *p* < 0.001).

### Treatment and survival

Of 667 patients, 331 (49.6%) had not received any type of treatment before surgery, whereas prior radiation of the affected spine segment had been conducted in 64 patients (9.6%) at a different institution. These patients with prior spinal radiation were significantly older by 8 years (73 vs. 65 years; *p* = 0.001) and the surgical complication rate was significantly higher for them (21.7% vs. 8.2%; *p* = 0.024).

Finally, 272 patients (40.7%) had received some form of systemic therapy or radiation of the primary cancer site before surgery.

Table 2 lists surgical procedures and median preoperative KPS. The majority of patients (n = 464; 69.6%) received a posterior instrumentation with decompression, 172 of which (37.1%) underwent an early additional vertebral body replacement before initiation of the adjunct treatment. A cement-augmentation of pedicle screws was conducted in 75 patients (11.2%). The number of patients with SMs originating from prostate cancer was 42 (50.6%) in the decompression only group and thus significantly higher proportioned than in the stabilization group (n = 93; 20.0%) and the kyphoplasty group (n = 10; 8.3%; Chi-square *p* < 0.001).

**Table 2:** Proportions of surgical techniques.

Surgical procedure		N (%)	Median preoperative KPS	Median postoperative KPS	P
Cases		667 (100)	70	80	0.004
Posterior instrumentation and decompression		292 (43.8)	70	70	0.540
Dorsoventral instrumentation with vertebral body replacement		172 (25.8)	80	80	0.412
Posterior decompression only		83 (12.4)	60	70	0.817
Kyphoplasty		120 (18.0)	60	80	** < 0.001**

*N* number, *KPS* Karnofsky performance scale score, *P* one-way ANOVA *p*-value.

The preoperative KPS scores were significantly different between surgical procedures (one-way ANOVA *p* = 0.012). The mean preoperative KPS score was significantly higher in the instrumented subgroup (KPS 73) compared to the decompression group (KPS 69; *p* = 0.038) and the kyphoplasty group (KPS 69; *p* = 0.013). The postoperative KPS increased significantly for the entire cohort (mean 73; median: 80; *p* = 0.004) and the kyphoplasty subgroup (mean: 77; median: 80; *p* < 0.001).

**Figure 2:**
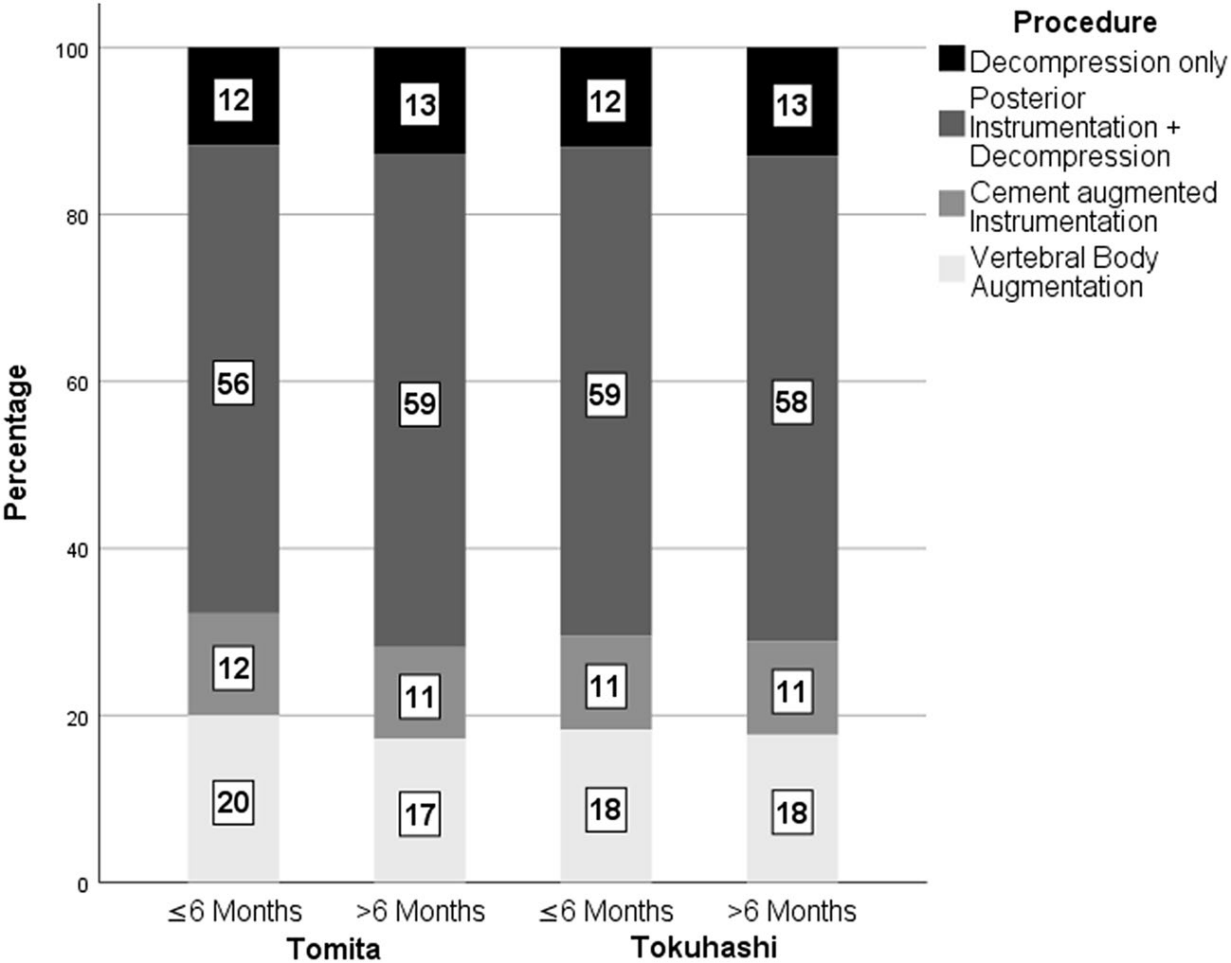
Proportions of surgical procedures performed in subgroups of Tomita and Tokuhashi scores for unfavorable and favorable prognosis, respectively.

The preoperative composite scores did not significantly influence decision-making as to which surgical strategy was chosen: the distribution of surgical procedures was not significantly different between stratified subgroups of favorable and unfavorable prognostic scores (Fig. 2).

Adjunct radiation and chemotherapy had been initiated in 71.2% during follow-up.

**Figure 3:**
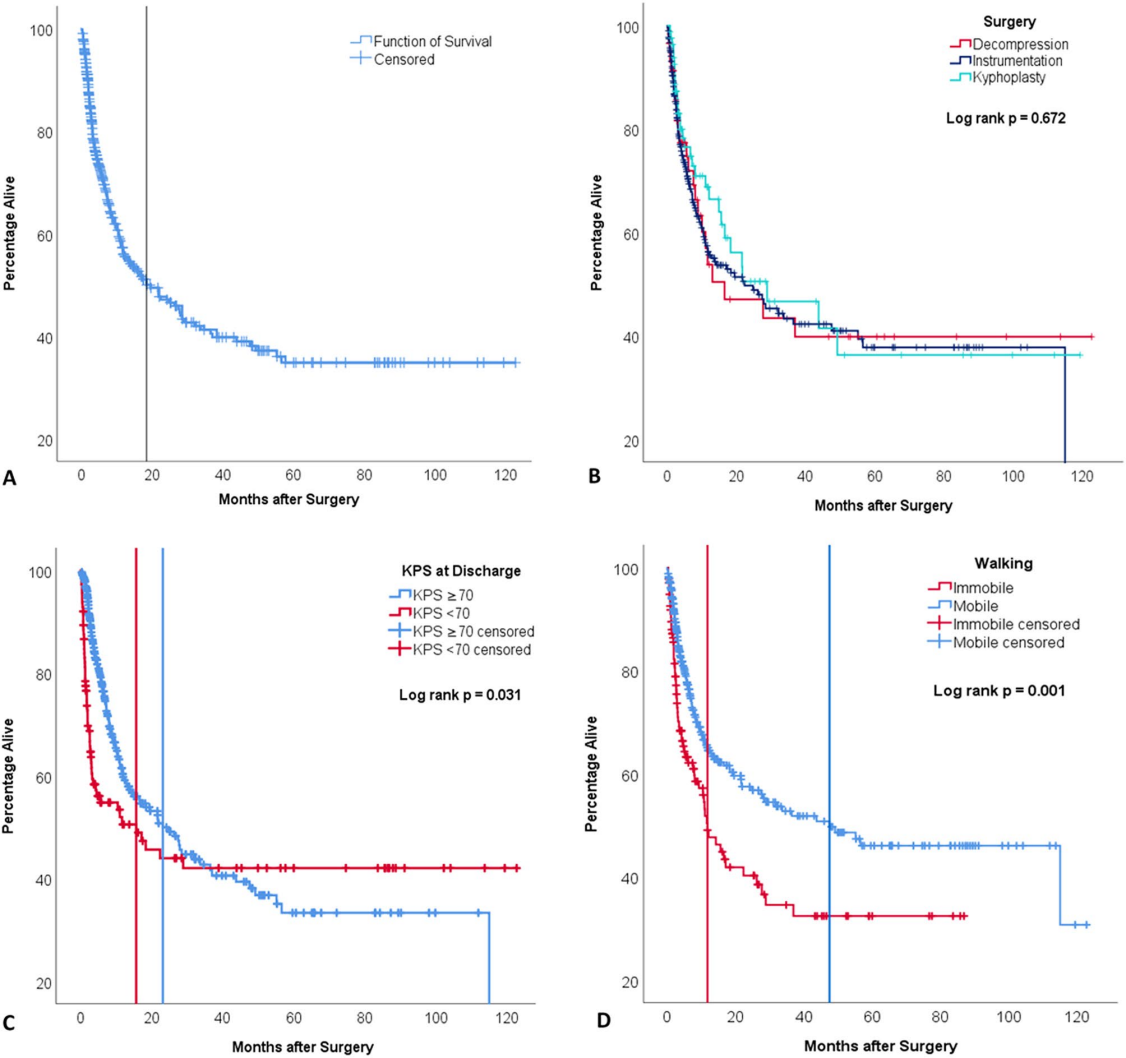
Kaplan–Meier estimation models of overall survival for the entire cohort **(A)**, stratified by surgical procedure performed **(B)**, functional status as per Karnofsky Performance Score at discharge **(C)** and ability to walk without aid by discharge **(D)**. Log rank test of statistical differences between means of subgroups performed for **(B,C,D)**. Vertical lines in **(A,C,D)** representing median overall survival. *KPS* Karnofsky Performance Scale score.

A Kaplan–Meier estimation of OS for the entire cohort is depicted in Fig. 3A. Median OS after surgery amounted to 18.4 months (95%CI 9.8 – 26.9). At 12 months after surgery, 56.0% of patients were alive. Stratification by surgical procedure revealed no significant difference in OS (log rank *p* = 0.672; Fig. 3B). After stratification by KPS at discharge, a significantly increased median OS of 23.1 months (95%CI 15.0 – 31.4) was revealed for the high KPS subgroup in comparison to the low KPS subgroup (15.6 months; 95%CI 0.5–30.8; log rank *p* = 0.031; Fig. 3C). After stratification of the cohort by their ability to ambulate by discharge, there was a significantly longer OS for the mobile subgroup (median OS 47.5 months; 95%CI 21.0–74.1) compared to the immobile subgroup (median OS 11.8 months; 95%CI 7.1–16.5; Fig. 3D). There was no significant difference in OS between patients with neurological deficits on admission and those without (neuro deficit: 57.0 months; no deficit: 57.2 months; *p* = 0.347). Patients who had received preoperative spinal radiation therapy did not have a significantly different OS in comparison to those without (*p* = 0.698).

In the Cox regression model, we found several significant predictors of the overall survival function with the preoperative KPS (*p* < 0.001), the KPS at discharge (*p* < 0.001) and the revised Tokuhashi score (*p* = 0.008), while the SINS (*p* = 0.325) and Tomita score (*p* = 0.079) missed statistical significance. The presence of visceral metastases in locations other than the spine (*p* = 0.019) significantly reduced OS.

A multiple regression analysis was run to predict KPS at discharge in relation to independent variables. The models revealed only age to have a significant impact on the KPS at discharge (*β* = − 0.1; *p* = 0.007; Table 3).

**Table 3:** Multiple linear regression of KPS at discharge and after 12 months in relation to various independent variables, controlled by preoperative KPS.

Independent variables	KPS at discharge	
	β	P
Age	**− 0.1**	**0.007**
Sex	− 0.9	0.359
Surgical procedure	1.0	0.063
No. of surgeries	− 1.8	0.330
Cancer of origin	0.1	0.630
Location of SM	− 0.0	0.985

*P* level of significance, *No.* number, *SM* spinal metastasis.

Bold type indicates statistically significant predictors.

Postoperative neurological deterioration was a significant independent predictor for increased mortality (OR 1.841; *p* = 0.027) in the logistic regression analysis, while age (*p* = 0.312), surgical procedure (*p* = 0.924), the cancer of origin (*p* = 0.836), the affected spinal segment (*p* = 0.161) or the occurrence of complications (*p* = 0.090) did not add significantly to the model (Table 4).

**Table 4:** Binomial logistic regression of mortality during follow-up.

Independent predictors for surgical complications	P	β		95% confidence interval	
Age	0.364	0.987		0.960	1.015
Surgical procedure type	** < 0.001**	**0.254**		**0.089**	**0.725**
Primary cancer	0.996	2.183		0.685	6.962
Spinal segment	0.355	–		–	–
Postoperative neurological deterioration	0.190	0.471		0.153	1.452

Independent predictors for mortality	P	β		95% confidence interval	
Age	0.312	0.991		0.975	1.008
Surgical procedure type	0.924	0.947		0.523	1.716
Primary cancer	0.836	1.907		0.419	1.802
Spinal segment	0.161	1.638		0.435	6.164
Postoperative neurological deterioration	**0.027**	**1.841**		**1.072**	**3.160**
Surgical complications	0.090	0.527		0.251	1.105

*P* level of significance. *β* regression coefficient.

Bold type indicates statistically significant predictors.

### Complications

Overall, 58 patients (8.7%) underwent 62 revisions for surgical complications (Table 5). Epidural rebleed with resulting neural compression occurred in 28 cases (4.2%), followed by hardware failure or screw malplacement in 12 (1.8%), wound infection in 10 (1.5%) and cerebrospinal fluid fistula in 8 cases (1.2%). Cement leakage was found in 4 patients (0.6%). No periprocedural deaths occurred.

**Table 5:** Proportions of surgical complications needing revision surgery, total and stratified by initial procedure type.

Surgical complications N cases (% of total)	Total	Decompression	Instrumentation	Kyphoplasty	P
	58 (8.7)	21 (3.2)	28 (4.2)	9 (1.4)	< *.001*
Epidural hematoma	24 (3.6)	13 (5.7)	10 (1.5)	1 (0.2)	
Screw malplacement	12 (1.8)	–	12 (1.8)	–	
Wound infection	10 (1.5)	5 (0.8)	3 (0.5)	2 (0.3)	
Cerebrospinal fluid fistula	8 (1.2)	3 (0.5)	2 (0.3)	3 (0.5)	
Cement leakage	4 (0.6)	–	1 (0.2)	3 (0.5)	

In the binary logistic regression, only the type of surgical procedure significantly influenced the occurrence rate of complications (*p* < 0.001). Patients in the decompression subgroup exhibited a significantly higher complication rate (n = 21; 25.3%) than the instrumentation subgroup (n = 28; 6.0%; *p* < 0.001) and the kyphoplasty subgroup (n = 9; 7.5%; *p* < 0.001; Table 5).

## Discussion

### Results

Our study encompassed 989 consecutive surgeries in 667 patients and, to our knowledge, at this time represents the largest single-center study covering a cohort of surgically managed patients with SMs. Our OS of 23.1 months in the high KPS subgroup was higher than in similarly structured series stratified by functional performance, with 16.0 months reported by Vanek et al., 13.0 months by Padalkar et al., 15.0 months by Park et al. and 13.0 months by Crnalic et al.^25–28^ The majority of studies report significantly lower OS times with worse preoperative Frankel grades, lower KPS and inability to walk, which is in concordance with our data indicating a shortened OS with a postoperative KPS less than 70^28–33^. Postoperative functional outcome as gauged by the median KPS increased after surgery, partly due to improved symptom control, and there was a significant increase in the proportion of patients with a KPS equal to or above 70. In addition, improvement of preoperative complaints was achieved in almost 90% of patients already during the postoperative in-patient stay, which is a crucial prerequisite for rapid initiation of adjunct radiation and systemic therapy. The supposedly more invasive circumferential stabilization procedures did not negatively impact on postoperative KPS or OS.

### Treatment strategy

**Figure 4:**
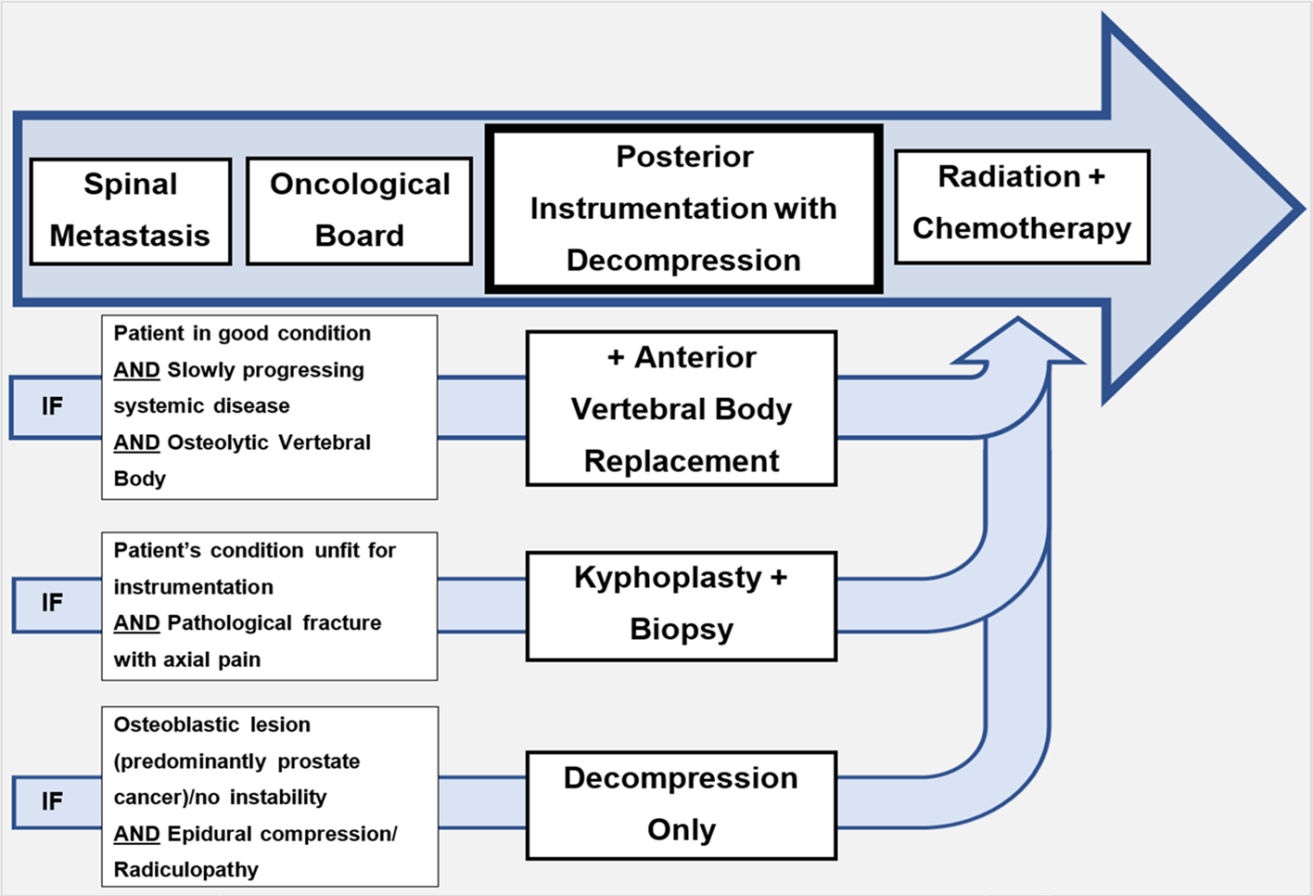
Algorithm for surgical management of patients diagnosed with a SM. Statistical analysis software: IBM SPSS Statistics for Windows v. Version 25.0 (Armonk, NY, 2017). https://www.ibm.com/products/spss-statistics.

This study is based on a consecutive cohort for which the decision-making has deliberately not been based solely on scores: any patient with symptoms corresponding to the SM was considered for posterior instrumentation and decompression first, or other procedures in selected cases. Analogously, adjuvant radiation and systemic therapy were offered to every patient, unless a palliative strategy had already been adopted or irradiation had been conducted too recently. Founded by these principles, we conceived a treatment algorithm for the choice of an appropriate surgical strategy and adjuvant therapy at our institution, which follows a comprehensive assessment of the patients’ current status and symptoms as well as characteristics of the primary cancer (Fig. 4). The algorithm has been closely adhered to for the patient cohort reported in this manuscript, yielding not only excellent improvements of the patients’ preoperative complaints, but also favorable oncological results.

Principally, any decision-making for the surgical management of SMs needs to strike a balance between adopting a treatment regimen that is too restrained and one that is too invasive, which may jeopardize the patient’s timely reconstitution and fitness for early adjunct treatment. The recommendations on invasive surgical management range somewhat throughout literature, with some authors primarily proposing sole en bloc spondylectomy similar to the paradigm for primary bone tumors^21,24,34–37^. Several authors generally posit that separation of tumor from the anterior neural compartment within the spinal canal alleviates postoperative stereotactic radiosurgery, which is not facilitated by simple laminectomy^22,23,38,39^. In comparison to a staged approach with anterior epidural decompression via an anterior approach, posterior spondylectomy may be accompanied by higher rates of postoperative morbidity and long-term compromise of biomechanical stability^40–43^. By contrast, we maintain that the primary focus of surgical management should be the preservation of neurological function and biomechanical stability rather than radical cytoreduction, thereby laying the foundation for adjunct therapy regimens that aim for oncological control. This may be achieved by tailoring the decompressive measures to the source of neural compression^36^. The separation technique and staged vertebral body replacement with anterior decompression are thus similar in concept, but different in execution. One might argue that the preference of one over another should be at the discretion of the operating surgeon, since there is no clear evidence for the superiority of any these techniques compared to the other, as long as neural decompression is achieved thoroughly.

It is debatable whether this paradigm of a consistent surgical concept is warranted in every case with an SM, yet highlighting the lack of level I evidence for the more invasive surgical strategies and in light of the patients’ need for long-term stability, functional preservation and symptom control, following a proactive surgical treatment paradigm appears more than justified.

This deliberately proactive surgical philosophy may be viewed as rather excessive when taking into account the subgroup of poor surgical candidates; this dilemma however may be addressed by a strategy of instrumentation in stages. Allowing time to recuperate from the posterior approach and reassessing the patient’s condition, fitness for additional vertebral body replacement as well as need for rapid initiation of adjunct therapy represented our paradigm of a truly *tailored* surgical management with the patients’ functional independence first in mind. We were thus able to collect a sizable number of surgical procedures, which allowed our surgeons to accomplish a substantial degree of routine expertise with various surgical appliances, i.e. the navigation system, cement-augmentation, minimally-invasive systems, circumferential stabilization with vertebral body replacement and, most recently, the implementation of carbon-fiber-reinforced pedicle screw systems. All together, these technical adjuncts serve to optimize and streamline surgical management by reducing surgical times, rates of wound breakdown as well as the length of in-hospital stay, although the carbon-fiber systems are still lacking in scientific evaluation of their efficacy and safety^11,44–46^. According to published data, these radiolucent systems bear important advantages over conventional systems for postoperative planning of adjunct radiotherapy and follow-up imaging, while offering ease of use and a safe risk profile^9,47–50^.

The resulting undelayed initiation of the subsequent therapy thus becomes a pivotal element within the framework of cancer treatment. In summary, any patient with a symptomatic SM who is not moribund retains eligibility for surgical treatment at our Institution. Those patients who suffer from a painful pathological fracture but are unfit to undergo instrumentation and decompression should be considered for a vertebral augmentation procedure, which is supported by high class evidence concerning its efficacy and safety (Fig. 4)^51^. Despite our deliberate non-reliance on scores, we however still maintain that the implementation of prognostic models should be further investigated to allow for more precise adjunct management by incorporating various outcome dimensions, as has been done by Cofano and colleagues^52^.

### Interdisciplinary management

The commitment to an interdisciplinary assessment, cooperation and joint treatment planning must be of the highest importance for any institution of cancer care. The management of a generally elderly population is challenged by a disease stemming from a multitude of primary cancers, which by definition is progressive and systemic when SMs are present. A tailored approach for the treatment of SMs gains relevance in the face of their increasing incidence and the continuously increasing life expectancy of cancer patients^8,53,54^. With perpetual advancements in oncological treatment regimens, it is crucial to continue investigating the outcomes after an interdisciplinary management.

The heterogeneity of the patient population with SMs challenges a multitude of specialists, who should adhere to a number of guiding principles for decision-making. An astonishing amount of prognostic statistical models and clinical decision rules have been developed within the recent two decades, integrating different aspects of clinical and radiographic nature^55–66^. These constructs suffer from two essential flaws: aside from often lacking external validation through high-level evidence, it is their inability to continually include novel treatments and progression of established modalities that might render them prematurely outdated. Most prognostic models are based on data collected on historical cohorts before the implementation of advanced surgical techniques, stereotactic radiotherapy and current pharmaceutical agents, which may explain their inaccuracy in predicting long-term survival as reported by Nater et al. and the Global Spine Tumour Study Group^57,67,68^. This notion bears particular importance in the ever-advancing field of Oncology^19–21,35,69–72^.

The complexity of oncological treatment and management of SMs in particular demands a routine interdisciplinary cooperation by a team of specialists involved in patient screening, evidence-based treatment and follow-up care. Several clinical decision rules have been proposed, such as the NOMS framework, OSRI and NICE guidelines amongst others^8,55–57,63,64,73,74^. These frameworks commonly integrate the patient’s condition, neurological status, primary cancer type and hallmarks of instability into an aid for deciding the appropriate treatment regimen. Due to insufficient evidence for superiority of one surgical technique over the other however, the guidelines often fail to delineate clear surgical strategies, delegating such decision-making to the individual surgeon^54,67^. This has generated a number of studies that apply vastly differing principles for surgical treatment, which hinders their comparability and impedes any consensus on a comprehensive surgical algorithm. All of these shortcomings and the shortsightedness of rigid prognostic score models immediately devalue the concept of the interdisciplinary management: as the key first step in SM treatment, an optimized and streamlined surgical strategy must pave the way for the patient’s fitness for adjunct therapy.

## Study limitations

We report on a retrospective surgically managed cohort, hence no comparison to non-surgical regimens should be made. Further, the function of overall survival encompasses a large cohort of patients with considerably diverse primary entities, which in itself dictates adjuvant treatment options and their efficacies.

The surgical treatment is complemented by a precisely tailored adjunct radiation, which essentially informs postoperative long-term tumor control. The evaluation of different radiotherapy concepts and protocols would however extend far beyond the scope of this study, which saw the surgical strategies as its focus. The lack of differentiated adjunct radiotherapy protocols in our results should be a clear limitation of this paper, causing considerable heterogeneity for the cohort.

## Conclusion

Surgical management of SMs with posterior instrumentation and decompression tailored to the given pathomorphology should generally represent the first step of treatment, the wide heterogeneity of the patient population requires a conclusive treatment strategy promoted by an interdisciplinary team. The need to achieve long-term symptom control and biomechanical stability stems from the growing life expectancy of patients with SMs, and requires a staged invasive surgical strategy currently not supported by level I evidence.

### Importance of the study

The surgical treatment of patients with spinal metastases (SM) continues to gain relevance in the wake of an ever-growing life expectancy of patients. While historically relegated to cases with acute neurological impairment, a landmark study from 2005 has since shown unparalleled benefits of a surgical treatment for the patient’s functional autonomy and mobility. The unmistakeable need for symptom control and functional competence lies the foundation for any adjunct therapy regimen, which strains patients’ physical health. Studies so far have been unable to provide a truly comprehensive surgical strategy and results on outcomes, due to the heterogeneity of the underlying disease, of the individual biomechanical hallmarks and the variability of surgical approaches. We were able to recruit the single largest cohort of patients with SMs managed according to our surgical paradigms and present a uniform treatment algorithm incorporating a variety of criteria.
